# End-of-Life Antibiotherapy: Factors Associated With Prescription

**DOI:** 10.7759/cureus.31634

**Published:** 2022-11-18

**Authors:** Elsa Campoa, Júlio Teixeira, Paulo Luz, Joana Magalhaes

**Affiliations:** 1 Medical Oncology, University Hospital Center of Algarve, Faro, PRT; 2 Medical Oncology, Universidade Lusófona's Research Center for Biosciences and Health Technologies (CBIOS), Lisbon, PRT

**Keywords:** supportive care, palliative care, end of life, cancer patient, antibiotherapy

## Abstract

Introduction

Most cancer patients spend their last days of life in the hospital, often receiving invasive and non-palliative interventions. These patients are particularly susceptible to infections, which are a major cause of death. The decision to use antimicrobials in a palliative context is difficult, given the lack of guidelines.

Objectives

To characterize patients who received antimicrobials at the end of life and analyze factors associated with their prescription decisions.

Methods

A retrospective analysis of patients who died in the Medical Oncology Service from January to December 2017 was done. In addition, the use of antibiotics in the last 15 days of life was considered. Clinical, therapeutic, and Eastern Corporative Oncology Group Performance Status Scale (ECOG PS) variables were analyzed using SPSS v23.

Results

There were a total of 116 deaths, of which 48.3% (n = 56) received antimicrobials in their last 15 days of life. The median age of the patients was 64.5 years. Most patients (55.4%) had an ECOG PS 4, of which 82.1% were stage IV tumors. The most frequent tumors were colorectal adenocarcinomas (21.3%), digestive non-colorectal (predominantly gastric or esophageal adenocarcinomas) (20.5%), and invasive breast carcinomas (16.4%). Asthenia (33.6%) and dyspnea (19.8%) were the main complaints, and most patients (55.4%) had respiratory infections. Fever was present in 51.8% of patients on antibiotics and was related to their use (p < 0.001). The use of antimicrobials is also related to higher C-reactive protein (CRP) values (p = 0.015).

Conclusions

The decision to institute antimicrobials at the end of life is related to clinical and analytical aspects suggestive of infection without considering the patient's general condition and the oncological disease's prognosis. Deprescription of antibiotic therapy is not yet a current clinical practice.

## Introduction

In recent years, there has been significant progress in ​​palliative care (PC) in Portugal in developing intrahospital, community teams, and health professionals' training in this area. Although indicated for all patients with chronic, progressive, and incurable diseases, most patients being followed up by the teams are cancer patients (CP) [1]. The use of antibiotic therapy in this population is quite frequent. Previous studies have reported their use in 87% of patients with advanced cancer who died in the hospital [1]. Other studies have presented similar data, with approximately 90% of patients receiving antimicrobials in the last week of life [2,3]. The prescription decision must be made after assessing the various factors involved. Their use is considered beneficial when symptomatic relief or an increased chance of survival is expected [3]. On the other hand, their harmful effects must be analyzed. Clinicians are currently facing difficulties in judging the clinical benefit to the patient since the most common complications in terminally ill patients are infections and fevers [3]. Several studies have been carried out regarding the use of antimicrobials in the context of PC. In a retrospective study with 141 patients, Thompson AJ et al. found that 86.9% received antibiotic therapy, of which 69.8% had clinical signs and/or symptoms suggestive of infection (mainly fever) [1]. Other authors analyzing 3,884 patients who died in PC concluded that 27% underwent at least one antimicrobial course in the last seven days of life, of which only 15% were diagnosed from complementary exams with infectious disease [4]. In the review by Macedo F et al., [3] the authors identified that the most frequent infectious foci in end-of-life patients are urinary and respiratory.

In a study with 255 terminally ill patients, White PH et al. [5] opted to perform antibiotic therapy restricted to patients with symptomatic infections (presence of fever, dyspnea, dysuria, or pain), with consideration being given to whether or not to administer antibiotic therapy to older patients with worse performance status (PS), not detecting differences in survival in groups with or without antibiotic prescription. Similarly, Reinbolt RE et al. considered the presence of symptoms, such as fever, cough, disorientation, dyspnea, dysuria, urinary frequency, hypotension, odynophagia, pain, skin changes, and sputum suspicion for infection [6]. Their results demonstrated that the use of antimicrobials and/or the presence of the infection has a negative relationship with survival. The benefit of symptomatic relief occurred in UTIs, not being seen when it came to respiratory or cutaneous infections. Brown NK and Thompson DJ [7] reported an opposite finding; during episodes of fever with suspected infection, patients who underwent antibiotic therapy showed a significant decrease in mortality (9% vs. 59%). Infections are one of these patients' leading causes of death, so the decision to prescribe antimicrobials in a palliative context is difficult for the clinician, especially given the lack of guidelines. The main objective of the present study is to characterize the patients who received antimicrobials in a group of CP who died during their hospital stay. As a secondary objective, we intended to analyze the variables that may influence the decision to prescribe an antibiotic at the end of life.

## Materials and methods

The study included all patients admitted to the Medical Oncology Department of Algarve University Hospital Center, Portugal, during the period from January 1 to December 31, 2017, who had the following criteria: 1) ≥18 years old at the date of diagnosis; 2) died during hospitalization; and 3) performed antimicrobials in the last 15 days of life. Information was collected from clinical files, including personal data (sex, age, and general condition), clinical data (main complaint, symptoms of dyspnea and fever, type of infection, oncological diagnosis, disease stage, and length of hospital stay), laboratory data (analytical values of hemoglobin, leukocytes, and CRP), and therapeutic data (ongoing cancer therapies, antibiotic class, and their length and therapeutic changes). In addition, the general condition of the patients was assessed using the Eastern Corporative Oncology Group Performance Status Scale (ECOG PS). The presence of symptoms and antimicrobial treatment was considered within the 15 days preceding death. The diagnosis of infection was made according to clinical judgment, mainly through the presence of symptoms associated with a particular focus and/or fever. Regarding types of infection, these were classified into four groups: without focus, respiratory, genitourinary, and intra-abdominal. Variables were analyzed using the version 23 SPSS statistical program. Continuous variables are presented as mean with SD or the median. The Chi-square test was used to compare proportions, and the t-test was used to compare continuous variables. In the analysis of statistical significance, p-values equal to or less than 0.05 were considered significant. This study was approved by the Institution's Ethics Committee for Health, and written consent was waived.

## Results

During the analyzed period, 116 deaths occurred, 21.6% (n = 25) of which were after transfer to the PC Unit. In 48.3% (n = 56) of patients, antibiotics were administrated in the last 15 days of life. The group of patients who underwent antibiotic therapy, with no differences in their characteristics in relation to the whole group, was predominantly female (57.1%), with a median age of 64.5 years. More than half (55.4%) had ECOG PS 4, of which 82.1% had stage IV tumors. The global characteristics of the population and each of the groups are shown in Table 1.

**Table 1:** General characteristics of the patients. NA: Not assessed; ECOG PS: Eastern Corporative Oncology Group Performance Status Scale.

	n (%)	n with antibiotics	n without antibiotics	P-value
Gender				0.342
Male	55 (47.4)	24	31	
Female	61 (52.6)	32	29	
ECOG PS				0.559
0	1 (0.9)	0	1	
1	3 (2.6)	1	2	
2	12 (10.3)	5	7	
3	44 (37.9)	19	25	
4	56 (48.3)	31	25	
Tumor stage				0.341
I	1 (0.9)	0	1	
II	3 (2.6)	1	2	
III	2 (1.7)	2	0	
IV	96 (82.9)	46	50	
NA	14 (12.1)	4	7	
Cancer treatments				0.373
Yes	52 (44%)	27	24	
No	65 (56%)	29	36	
Symptoms at admission				0.461
Non-specific				
Prostration / asthenia	39 (33.6)	17	22	
Respiratory				
Dyspnea	23 (19.8)	12	11	
Gastrointestinal				
Dysphagia / refusal to eat	3 (2.6)	2	1	
Abdominal pain	6 (5.2)	1	5	
Nausea / vomiting	7 (7.0)	3	4	
Neurological				
Change of consciousness	9 (7.8)	4	5	
Change in muscle strength	9 (7.8)	5	4	
Others	9 (7.9)	3	6	
Average hospitalization length				0.401
<= 5 days	39 (33.6)	16	23	
6-10 days	31 (26.7)	16	15	
10-15 days	14 (12.1)	9	5	
16-20 days	15 (12.9)	6	9	
>20 days	17 (14.6)	9	8	

In the whole group, the most frequent tumors were colorectal adenocarcinomas (21.3%), digestive non-colorectal (predominantly gastric or esophageal adenocarcinomas) (20.5%), and invasive breast carcinomas (16.4%) (Table 2). In addition, six patients had synchronous tumors.

**Table 2:** Primary cancer diagnosis.

	N (%)
Breast	20 (16.4%)
Digestive colorectal	26 (21.3%)
Digestive non-colorectal	25 (20.5%)
Genitourinary	13 (10.6%)
Head and neck	10 (8.2%)
Gynecological	10 (8.2%)
Cutaneous	3 (2.5%)
CNS	5 (4.1%)
Others	10 (8.2%)
Total	122 (100%)

A higher number of patients had metastatic disease (82.1%), predominantly liver metastasis (37.9%), pulmonary (31.9%), bone (29.3%), nervous system (10.3%), or others. Six patients had synchronous tumors. Among the whole group, the main complaints relating to hospital admission were prostration and asthenia (33.6%) and dyspnea (19.8%). Hospitalizations had an average length of 13.9 days (SD = 14.4), with 33.6% being short hospitalizations (≤ 5 days).

In the last 15 days of life, 28.4% (n = 33) had a fever. There was an administration of antibiotics due to the presumptive diagnosis of infection in 48.3%, predominantly of respiratory focus (55.4%), followed by genitourinary (19.6%), unfocused (19.6%), and intra-abdominal (5.4%). Analytically, with a mean hemoglobin value of 10.7 g/L, the leukocyte value was 15,814 L-1, and the PCR value was 151.4 g/dL. In the whole group, antibiotics were administrated in 48.3% of patients. When analyzing patients with a fever in the last 15 days of life, antibiotics were administered to 96.4% of patients.

Only 44% (n = 51) of the patients were under active cancer treatment. The remaining 56% (n = 65) were on supportive therapy. Of the patients on supportive therapy, 66.2% had never undergone cancer treatment (chemotherapy, radiotherapy, or target therapies). Before administering antibiotics, culture tests were carried out, namely blood cultures in 22.4% and urine cultures in 19.8% of patients, of which 0.9% and 6.9%, respectively, were positive. The administrated antibiotics were predominantly beta-lactam with beta-lactamases inhibitors (53%) and 2nd and 3rd-generation cephalosporins (18%). The median length of antibiotic therapy was seven days (AIQ 5), with a duration of 7-8 days in 53.6% of therapies. In 5.7% of cases, the antibiotic therapy duration was extended. In contrast, it was found that 37.6% of patients had <7 days of antibiotic therapy, and 30.4% had ≤3 days of antibiotic therapy. Patients with the shortest duration of antimicrobial therapy (30.4%) were also those who had shorter hospital stays (r = 0.595, p <0.001).

Among the factors related to antibiotic prescription, there is a relationship with the presence of fever (r = 0.500, p < 0.001). This factor proved to be a criterion used by clinicians for the institution of antibiotic therapy, regardless of the focus, since there were no differences between the various likely foci of infection. In addition to fever, other clinical factors, such as the presence of symptoms (namely dyspnea) or the increase in inflammatory parameters, were also considered, highlighting the positive relationship with the CRP values ​​(r = 0.228, p = 0.015). For higher CRP values, there was an increase in the frequency of antibiotic administration. Table 3 presents an analysis of factors associated with the institution of antibiotic therapy. Compared to the administration and not to antibiotic therapy, it is noted that most patients had a fever.

**Table 3:** Comparative data with or without the use of antibiotics. ATB: Antibiotic.

	N with ATB (n=56)	N without ATB (n=60)	P-value
Fever (last 15 days of life)			
Yes	29	4	<0.01
No	27	56	
Dyspnea	12	11	0.680
Type of infection			
Without focus	8	1	0.687
Respiratory	30	3	
Genitourinary	11	0	
Intra-abdominal	3	0	
Culture tests			
Hemocultures	24	2	<0,01
Urine cultures	22	1	<0,01

The presence of infection was primarily of a respiratory nature, although the symptom of dyspnea occurred at a similar frequency in both groups (with or without antibiotic therapy). In our study, factors associated with the patient, namely their general and functional status, assessed using the ECOG PS scale, revealed that the group was functionally impaired, with limitations in daily activities and requiring support from third parties, noted by the presence of ECOG PS 3 in 37.9% and ECOG PS 4 in 48.3% of patients, respectively. However, the patients' functionality indexes were not related to the administration of antibiotics (r = 0.149, p = 0.110). Likewise, factors associated with the disease, such as its stage, were not related to the administration of antibiotics (r = 0.083, p = 0.586).

Similarly, there were also no statistically significant differences in relation to the presence of active cancer treatments (r = 0.083, p = 0.377); that is, the decision regarding the measures to be taken with regard to antibiotic therapy did not differ between patients in active treatments for cancer disease and patients in supportive care.

## Discussion

In the period under analysis, there were 116 deaths, mostly with functional assessment of ECOG PS 4 and stage IV tumors. The most frequent primary locations were the colon and breast, which are the most common cancers [8]. The absence of lung tumors in this group of patients is associated with the institutional reality in which thoracic tumors are managed at the Pulmonology Department. In general, fever occurred in 28.4% of patients, and in the group of patients who underwent antibiotics, its frequency was around 50%, with a positive correlation (r = 0.500, p < 0.001). On the other hand, analyzing patients with fever, it appears that these <5% did not undergo antibiotic therapy. The use of antimicrobials is also related to higher CRP values (r = 0.228, p = 0.015). However, there is no relation between the administration of antibiotics and the ECOG PS (r = 0.149, p = 0.110), tumor stage (r = 0.083, p = 0.586), or presence of active treatments (r = 0.083, p = 0.377). These data will be analyzed below. 

From the first data presented, the small number of patients who were transferred to PC stands out (21.6%), which, due to the characteristics of the population, may be related to a late referral to PC. Previous studies have shown that the late referral of patients to PC is linked to earlier mortality with lower quality of life [9]. On the other hand, symptomatic control is one of the most valued aspects of PC by patients and family members [10]. The most frequent symptoms in this study agree with previous data that already presented fatigue and dyspnea as some of the main symptoms presented by end-of-life patients [11]. Regarding the primary data under analysis in this study, it should be pointed out that in the last 15 days of life, there was a presumptive diagnosis of infection in 48.3% of patients, predominantly of a respiratory nature. The high frequency of clinical suspicion of infection in end-of-life patients agrees with those already presented in previous studies regarding a high rate of suspected infection in these patients, associated with the high frequency of infectious processes in the palliative phase that often effectively determine death [12].

Half of the patients had presumed infection in the last days of their lives with antibiotic administration, being that almost all of them underwent antibiotic therapy. However, it was not possible to verify the purpose of their use, whether curative or palliative. These data are consistent with previous studies, which consider that the use of antibiotics in this context is quite frequent, with rates well above 87-90% in the last week of life [1-3]. However, other studies have lower frequencies with data that reflect the overuse of antimicrobials in this group of patients. Similar to the literature that already indicated documentation by culture tests in approximately 15% of cases, data in our group were still lower [4].

Regarding the factors associated with an antibiotic prescription, the presence of fever stands out, denoting a statistically significant correlation. On the one hand, of all the patients under antibiotic therapy, only 51.8% had a fever. On the other hand, of all patients with fever, antibiotics were not administrated to 6.7% of them due to an absence of considered benefits due to fever of non-infectious etiology. In fact, fever can have multiple etiologies beyond infection. It can be induced by the tumor itself, by drugs, or by thromboembolic events, thus making diagnosis difficult and leading to the overuse of antibiotics [12]. On the other hand, the benefit of antibiotic treatment in the presence of fever is reinforced in the literature, with symptomatic improvement and increased survival [7,13]. Other studies, in contrast, demonstrate a negative association between the use of antibiotics and the presence of infection with survival. Regarding the type of infection, in our study, the main infectious focus was respiratory. Other studies only report benefits in symptomatic relief in UTIs, in contrast to what occurs in respiratory or skin infections [6].

The presence of increased inflammatory parameters was another factor related to the institution of antibiotic therapy, namely CRP values. The increase in CRP in end-of-life patients can occur in the absence of an infectious process, presenting itself as a marker of poor prognosis. In clinical practice, increases in CRP, similar to our data, are interpreted as the presence of infection, and antibiotic treatment is started, even though most culture tests turn out to be negative afterward, and the patients die [14]. In contrast to the factors of "fever" and "CRP," which were related to antibiotic therapy, factors such as the general condition of the patient, the stage of his illness, or even the presence or absence of active treatments for the control of CD had no statistical relation to antibiotic administration. The patient's general condition is not related to the antibiotic prescription decision, considering that it does not interfere and is not a preponderant variable for the clinician. Few studies in the literature address the association of the patient's functional status with the institution of antibiotics. However, White PH et al. [5] presented conflicting data, emphasizing the importance of PS, demonstrating that in older patients with worse PS, antibiotics did not influence survival compared to patients to whom antibiotics were not administered.

The patient’s general condition is not related to the antibiotic prescription decision, considering that it does not interfere and is not a preponderant variable for the clinician. Few studies in the literature address the association of the patient’s functional status with the institution of antibiotics. However, White et al. [5] presented conflicting data, emphasizing the importance of PS, demonstrating that in older patients with worse PS, antibiotics did not influence survival compared to patients to whom antibiotics were not administered.

Also, the CD stage was not related to the administration of antibiotics (p = 0.586), and it is expected that patients in the early stages will have antibiotics administered with curative intent. Its deprescription may be considered in terminally ill patients according to the risk/benefit weighting. The lack of statistical relation in our data can be justified by most patients (82.9%) being in stage IV, with the distribution in the remaining numbers reduced so that it is possible to infer statistical differences.

Another analyzed factor, the presence or absence of active cancer treatments, did not show a statistically significant association with antibiotic prescription (p = 0.377). The literature shows that even in advanced stages, antibiotics should be prescribed if they lead to a clinical benefit, quality of life, or survival. However, therapeutic obstinacy should not be carried out [12]. Regarding the institution of antibiotics in patients with active treatments, namely target treatments with immune checkpoint inhibitors, studies have been the subject of attention since antibiotic administration seems to have harmful effects, with a decreased response to treatments and a reduction of progression-free time, without, however, influencing survival [15]. Nonetheless, it does not show any influence before the start of therapy [16].

Of the patients who underwent antibiotic therapy, 30.4% had a duration of less than or equal to three days due to the occurrence of death (r = 0.595, p <0.001). In this particular subgroup, it would be important to understand whether a clinical benefit with antibiotic administration would be expected or whether symptoms should be palliated and managed with other drugs or approaches, such as the use of opioids to control dyspnea or pain in the case of respiratory infections, the main source of infection in this study [17].

This study presents some limitations, such as a retrospective analysis and data from just one medical center with a small dataset. It is also challenging to address the clinical reason why the doctor prescribed antimicrobial therapy.

In view of all the factors exposed, it should be considered that in patients with PC, deprescription is already consensual in some areas of activity, with several norms associated with the discontinuation of therapies that do not present benefits in the control of symptoms/diseases. Regarding antibiotic therapy, the absence of guidelines makes this decision less consensual, dependent on the clinician's decision, and being considered in each patient/clinical situation [12].

The consensus data in several studies are related to the clinical benefit to symptomatic relief, improved quality of life, and increased survival. Thus, through these possibilities, antibiotic administration must be strongly considered to establish whether the benefits to be achieved outweigh the risks and adverse effects associated with the prescription.

## Conclusions

The rate of antibiotic administration in the last 15 days of life is relatively high, being carried out in most patients with a suspected infection. The presence of fever and increased inflammatory parameters was, nevertheless, associated with an antibiotic prescription, regardless of the patient's functional status, disease stage, or active cancer treatments. Thus, there is a need to alert clinicians to approach the patient as a whole and not just the measurable factors at the time of the decision to administer antibiotics, seeking their greatest benefit.

The present study is a retrospective study in which it was not possible to evaluate the curative or palliative purpose of the institution of antimicrobials. Therefore, it is essential to carry out future prospective studies to assess the therapy's intention and develop guidelines for decision-making.
